# Simultaneous regulation of ferroptosis suppressor protein 1 and glutathione peroxidase 4 as a new therapeutic strategy of ferroptosis for esophageal squamous cell carcinoma

**DOI:** 10.1007/s10388-022-00982-x

**Published:** 2022-12-28

**Authors:** Wataru Miyauchi, Yuji Shishido, Yoshiaki Matsumi, Tomoyuki Matsunaga, Masahiro Makinoya, Shota Shimizu, Kozo Miyatani, Teruhisa Sakamoto, Yoshihisa Umekita, Toshimichi Hasegawa, Yoshiyuki Fujiwara

**Affiliations:** 1 Division of Gastrointestinal and Pediatric Surgery, Department of Surgery, School of Medicine, Faculty of Medicine, Tottori University, 36-1, Nishi-cho, Yonago, Tottori 683-8504 Japan; 2 Division of Chemical Biology, Technical Department, Tottori University, Yonago, Japan; 3 Division of Organ Pathology, Department of Pathology, Faculty of Medicine, Tottori University, Yonago, Japan

**Keywords:** Esophageal squamous cell carcinoma, Ferroptosis, Lipid peroxidation, Cell death

## Abstract

**Background:**

Ferroptosis suppressor protein 1 and glutathione peroxidase 4 have been identified as key molecules in two independent pathways associated with ferroptosis inhibition. This study investigated the prognostic significance and clinical associations of FSP1 and GPX4 expression in esophageal squamous cell carcinoma (ESCC) and assessed the therapeutic potential of regulating these molecules in ESCC cells.

**Methods:**

Immunohistochemical analysis was performed on surgical specimens of 97 patients with ESCC for FSP1 and GPX4 expression. To identify the change in ESCC cell viability, FSP1 and GPX4 inhibitors were administered to three cell lines. In addition, ferroptosis as the cause of reduced cell viability by FSP1 and GPX4 inhibition was confirmed.

**Results:**

Prognosis was significantly worse for patients in the group positive for both FSP1 and GPX4 compared with the other groups (*p* < 0.001). In multivariate analysis, positivity for both FSP1 and GPX4 was an independent poor prognostic factor (*p* = 0.002). The combination of FSP1 and GPX4 inhibitors induced cell death more potently than each inhibitor did alone. Furthermore, the ferroptosis inhibitor markedly canceled this cell death.

**Conclusions:**

Overexpression of FSP1 and GPX4 is a poor prognostic factor for patients with ESCC. Simultaneous suppression of both FSP1 and GPX4 caused potent cell death, which was markedly abrogated by ferroptosis inhibitors. These findings indicate that simultaneous regulation of FSP1 and GPX4 may be a new therapeutic target in ESCC.

**Supplementary Information:**

The online version contains supplementary material available at 10.1007/s10388-022-00982-x.

## Introduction

Esophageal cancer is the sixth most common cancer globally, with more than 480,000 new cases annually [1]. Moreover, this cancer is the fifth leading cause of cancer-related mortality, with 400,000 deaths annually [1]. In addition, the 5-year survival rate after esophagectomy is only 55.6% [2]. Thus, esophageal cancer persists as one of the malignant tumors with a poor prognosis [2].

Inducing cancer cell death is an important strategy in chemotherapy. Chemotherapeutic drugs for esophageal cancer include 5-fluorouracil (5-FU), platinum, and taxane [3], all of which induce apoptosis [4]. Esophageal squamous cell carcinoma (ESCC) has an extremely high mutation rate of apoptosis-inducing p53 (> 90%) [5] and is known to be resistant to chemotherapy [6].

In 2012, researchers discovered ferroptosis, defined as cell death by iron-dependent lipid peroxidation reaction [7]. Research further showed that ferroptosis is a different mechanism of cell death than that which occurs from apoptosis and necrosis, suggesting that ferroptosis is able to induce cell death even in apoptosis-resistant cancer cells [7]. Glutathione peroxidase 4 (GPX4) was reported in 2014 as a factor that inhibits ferroptosis [8]. Since then, several reports have implicated GPX4 in cancer prognosis [9, 10]. Subsequently, in 2019, ferroptosis suppressor protein 1 (FSP1) was reported as a new ferroptosis inhibitor [11, 12], previously known as apoptosis-inducing factor mitochondrion-associated 2 [13]. Both GPX4 and FSP1 negatively regulate ferroptosis by inhibiting lipid peroxidation reactions through different pathways [8, 11, 12].

We previously reported that high expression of GPX4 correlated with worse prognosis in ESCC [14]. The present study aimed to determine the prognostic significance of FSP1 and its association with GPX4 in patients with ESCC. We used the same cohort and performed additional experiments with FSP1, which is associated with another ferroptosis inhibitory pathway. Furthermore, we also examined the therapeutic potential for ferroptosis inhibition with FSP1 and GPX4 in patients with ESCC.

## Materials and methods

### Patients and pathologic specimens

Study participants were 97 patients who underwent radical esophagectomy for ESCC at Tottori University Hospital, Yonago, Japan, from January 2009 to December 2017. Stored paraffin-embedded specimens were obtained for immunohistochemical analysis. Patients with multiple primary cancers were excluded. Pathological diagnosis was confirmed according to the Japanese Classification of Esophageal Cancer [15]. The patients with clinical T1N0 underwent surgery without preoperative treatment. Patients with ≥ T2 or with lymph node metastasis (cStage ≥ 2) received neoadjuvant chemotherapy (NAC), followed by esophagectomy. In principle, patients treated with NAC underwent surgery 5–7 weeks after NAC completion avoid the influence of NAC. As standard chemotherapeutic drugs, 5-FU and cisplatin were used for all eligible patients except those with impaired renal function, who were instead treated with 5-FU and nedaplatin. The standard surgical procedure was subtotal esophagectomy by a right thoracic approach with three-field lymphadenectomy and reconstruction using a gastric tube. The tumor samples were fixed in 10% neutral buffered formalin solution and embedded in paraffin.

### Immunohistochemical analysis

Immunohistochemistry was performed according to the standard protocols, which are further described in the Online Resource.

The expression of FSP1 was defined as < 20% negative and ≥ 20% positive in terms of the tumor-stained area. Immunolabeling was evaluated by three investigators (W.M., Y.S., and Y.U.); consensus was reached in all cases. To evaluate GPX4 expression, our previous staining results were used as follows [14]: “Expression of GPX4 and HMOX1 in the tumor was assessed on a 4-point scale based on the percentage of tumor cells with positive staining (immunohistochemistry) score: 0 [< 10%], 1 [10–50%], 2 [50–90%], 3 [> 90%]).” An immunohistochemistry score of ≥ 2 was considered a positive expression. Immunolabeling was evaluated by three investigators (W.M., Y.S., and Y.U.); consensus was reached in all cases.

### Cell lines and cell culture

Three human ESCC cell lines, including KYSE30, KYSE510, and KYSE520, were grown in Roswell Park Memorial Institute Medium supplemented with 10% fetal bovine serum. All cell lines were maintained under 37 °C in atmospheric air supplemented with 5% carbon dioxide and passaged at a ratio of 1:3–1:10 every 2–3 days. Cell line and reagent details are described in the Online Resource. Clinical information on these cell lines is summarized in Table S1 in the Online Resource.

### Western blotting analysis

In this analysis, the KYSE30, KYSE510, and KYSE520 cells were seeded at 2 × 10^5^ cells in four 6-cm dishes and incubated overnight. After 24 h, the cells were lysed and protein extracted. Detailed descriptions of the subsequent steps are included in the Online Resource.

### Cell proliferation assay after treatment with GPX4 or FSP1 inhibitors

ESCC cell lines were treated with iFSP1—an FSP1 inhibitor—and (1S, 3R)-RSL3 (RSL3; a GPX4 inhibitor) alone or in combination. Cell proliferation was assessed using the Cell Counting Kit-8 (CCK8) according to the manufacturer’s protocol, which is further detailed in the Online Resource.

### Cell death inhibition assay

The procedure and elapsed time from cell seeding, addition of drugs, and cell survival evaluation are the same as the cell proliferation assay. The added drug included a combination of iFSP1 and RSL3 plus liproxstatin-1 (Lipro-1, a ferroptosis inhibitor) or Z-VAD-FMK Caspase Inhibitor VI (Z-VAD, an apoptosis inhibitor) or Necrostatin-1 (Necro-1, a necrosis inhibitor), respectively. Detailed descriptions of the subsequent steps are included in the Online Resource.

### Statistical analysis

For overall survival and relapse-free survival, survival curves were estimated by Kaplan–Meier analysis; differences in survival curves were compared using the log-rank test. Cox proportional hazards models were used for univariate and multivariate analyses. All quantitative values are presented as the median. For FSP1 and GPX4, association between expression and patient's characteristics was examined using the chi-squared test and Fisher exact test for categorical variables. All *p* values < 0.05 were considered statistically significant. Statistical analyses were performed using GraphPad Prism (GraphPad Software, La Jolla, CA, USA) and SPSS version 25.0 (IBM, Armonk, NY, USA).

## Results

### FSP1 and GPX4 expression associated with ESCC prognosis

**Fig. 1:**
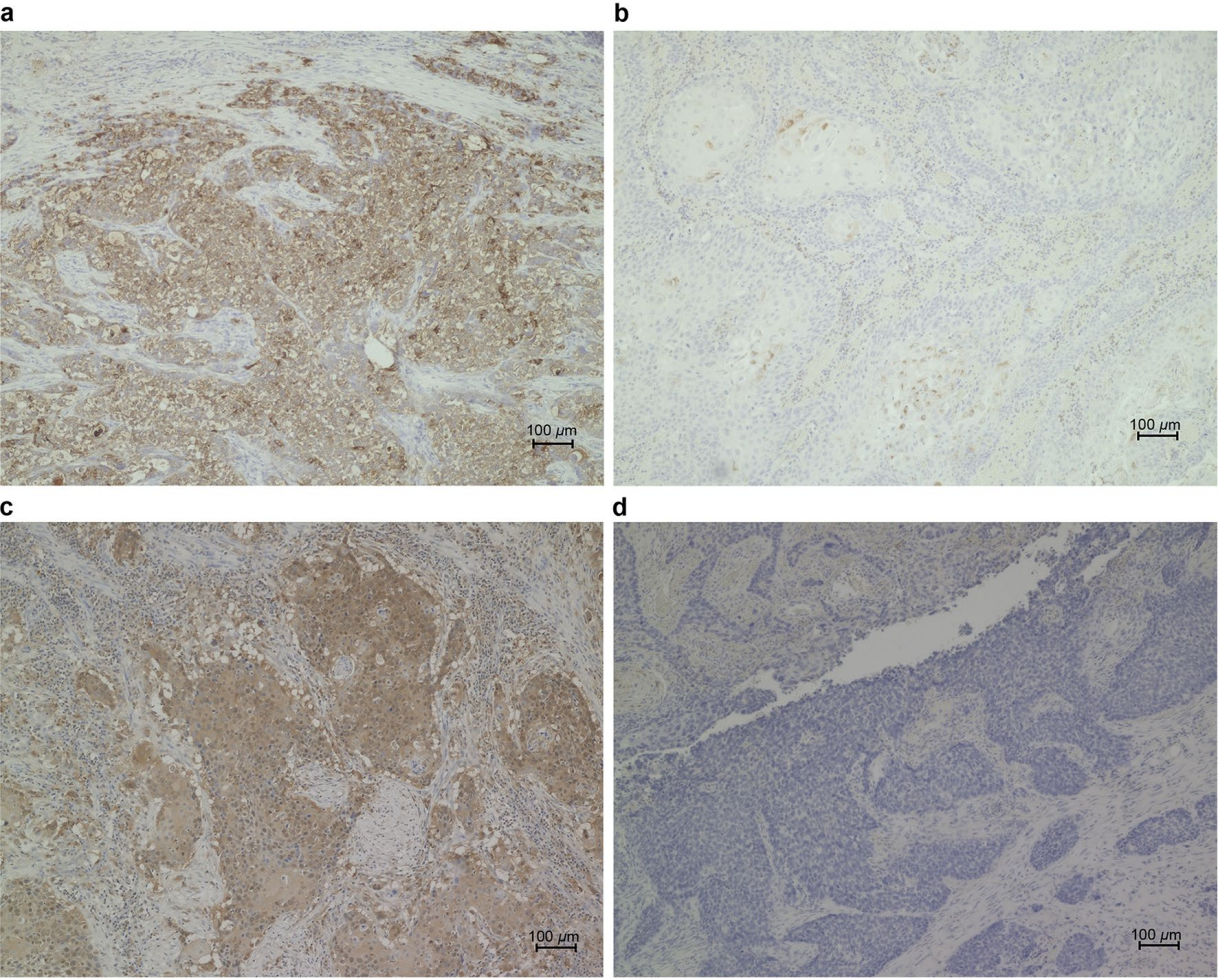
Representative immunohistochemical stains for FSP1 and GPX4 in patients with ESCC. Magnification × 100; scale bar, 100 μm. **a** High-expressing FSP1 ESCC. The FSP1 protein is localized in cytoplasmic regions. **b** Low-expressing FSP1 ESCC (FSP1-negative case). **c** High-expressing GPX4 ESCC. The GPX4 protein is localized in cytoplasmic regions. **d** Low-expressing GPX4 ESCC (GPX4-negative case). *ESCC* esophageal squamous cell carcinoma; *FSP1* ferroptosis suppressor protein 1; *GPX4* glutathione peroxidase 4

Expression of FSP1 and GPX4 in resected specimens from patients with ESCC was evaluated by immunohistochemistry and classified as positive or negative based on the percentage of positively tumor-stained area (Fig. 1).

Of the 97 patients, 20 (20.6%) were positive for FSP1 and 40 (41.2%) for GPX4. The FSP1-positive group had a significantly higher invasion depth (*p* = 0.001) and disease stage (*p* = 0.029) than the FSP1-negative group. The GPX4-positive group had significantly higher invasion depth (*p* = 0.014), lymph node metastasis (*p* = 0.001), lymphatic involvement (*p* = 0.019), vascular involvement (*p* = 0.011), and disease stage (*p* = 0.002) than the GPX4-negative group (Table 1).

**Table 1:** Patient characteristics stratified by FSP1 and GPX4 status

Characteristics	FSP1		*p* value	GPX4		*p* value
	Positive	Negative		Positive	Negative	
Age, years			0.400			0.167
< 70	14	46		28	32	
≥ 70	6	31		12	25	
Sex			0.294			0.301
Male	15	67		32	50	
Female	5	10		8	7	
Body mass index (kg/m^2^)			0.526			0.484
< 22	12	52		28	36	
≥ 22	8	25		12	21	
Tumor location			0.295			0.659
Upper/middle	11	52		27	36	
Lower	9	25		13	21	
Neoadjuvant chemotherapy			0.729			0.569
Absent	9	38		18	29	
Present	11	39		22	28	
Differentiation			1.000			0.321
Well/moderate	18	66		33	51	
Poor	2	11		7	6	
Invasion depth			0.001**			0.014*
pT1	3	43		13	33	
pT2/3/4	17	34		27	24	
Lymph node metastasis			0.213			0.001**
Absent	6	35		9	32	
Present	14	42		31	25	
Lymphatic involvement			0.385			0.019*
Absent	3	21		5	19	
Present	17	56		35	38	
Vascular involvement			0.198			0.011*
Absent	4	27		7	24	
Present	16	50		33	33	
Disease stage			0.029*			0.002**
0/1	2	27		5	24	
2/3/4	18	50		35	33	

*p < 0.05; **p < 0.01; ***p < 0.001

**Fig. 2:**
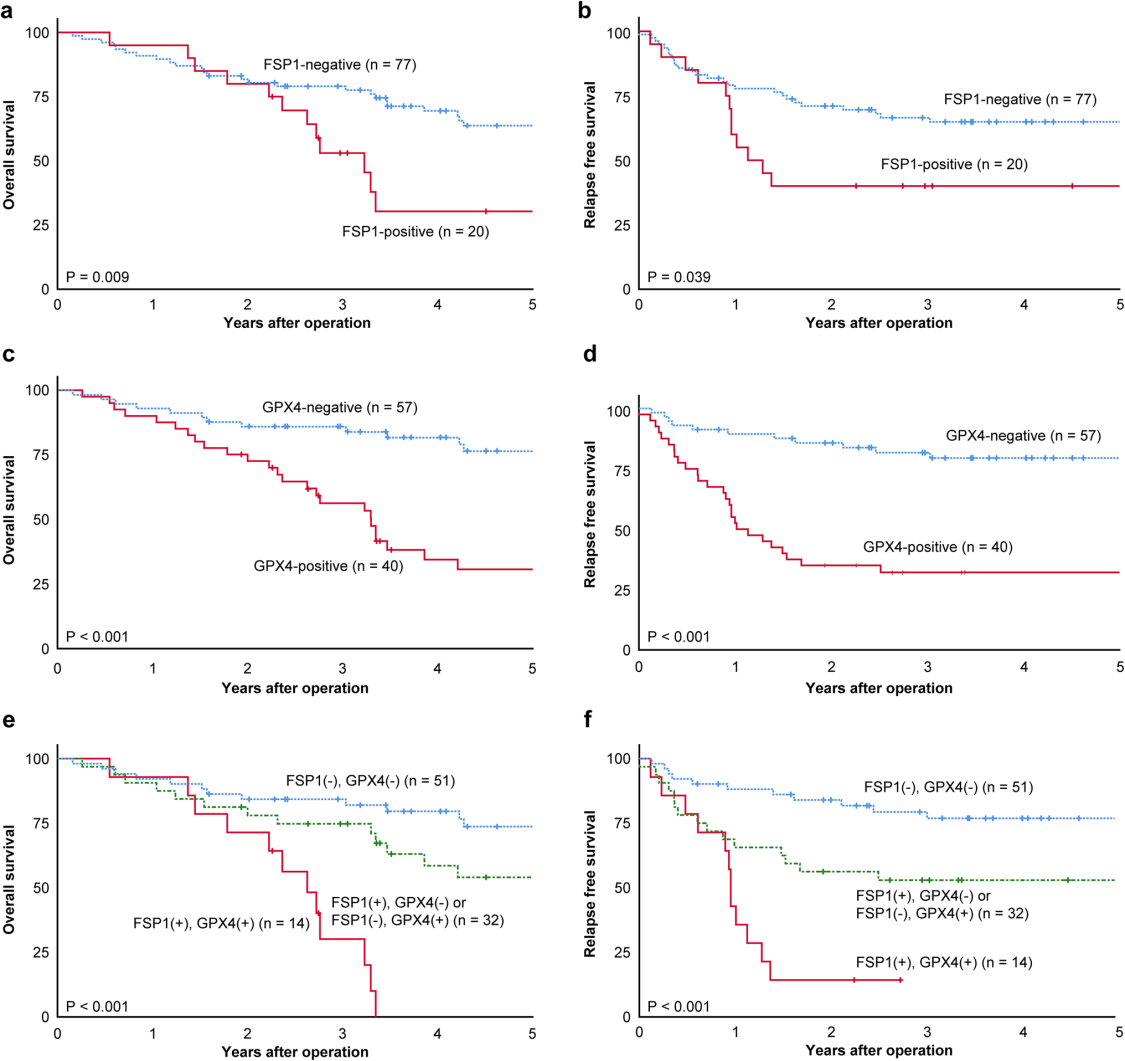
Kaplan–Meier survival curves categorized by FSP1 and GPX4 immunoreactivity in ESCC. **a**, **b** Survival based on FSP1 expression. **c**, **d** Survival based on GPX4 expression. **e**, **f** Survival based on simultaneous expression of FSP1 and GPX4. *ESCC* esophageal squamous cell carcinoma, *FSP1* ferroptosis suppressor protein 1, *GPX4* glutathione peroxidase 4

Next, we examined the correlation between FSP1 and GPX4 expression and prognosis of patients with ESCC. The FSP1-positive group had significantly worse overall survival and relapse-free survival than the negative group (Fig. 2a, b). Similar results were obtained for the GPX4-positive and -negative groups (Fig. 2c, d).

In addition, we compared the prognosis between three classified groups based on the combined expression patterns of FSP1 and GPX4. The breakdown of FSP1 and GPX4 positivity/negativity is shown below. Both FSP1- and GPX4-positive; *n* = 14, FSP1-positive and GPX4-negative; *n* = 6, FSP1-negative and GPX4-positive; *n* = 26, Both FSP1- and GPX4-negative; *n* = 51. Groups that were positive for only one of FSP1 or GPX4 were considered together in one group. The group with both FSP1- and GPX4-positive expression had a significantly worst prognosis (*p* < 0.001) (Fig. 2e, f). In contrast, the group with both FSP1- and GPX4-negative results showed the best prognosis compared with the other groups.

We also examined whether the combined FSP1 and GPX4 expression pattern was associated with NAC in patients with ≥ T2 or lymph node metastasis (Table S2). The prognostic comparison of the patients with ≥ T2 or lymph node metastasis among the same three groups categorized according to the FSP1 and GPX4 expression revealed a pattern that was similar to that observed in the entire patient population (Fig. S1). In multivariate analysis, positivity for both FSP1 and GPX4 was an independent poor prognostic factor (*p* = 0.002) (Table 2). For FSP1 and GPX4 expression, both positive groups, which had a particularly poor prognosis, were used as one of the covariates in the multivariate analysis.

**Table 2:** Univariate and multivariate analyses of clinicopathological factors influencing overall survival

Variables	Univariate analysis			Multivariate analysis		
	HR	95% CI	*p* value	HR	95% CI	*p* value
Age, years (< 70 vs. ≥ 70)	0.693	0.361–1.330	0.270			
Sex (male vs. female)	1.636	0.643–4.158	0.301			
Body mass index (< 22 vs. ≥ 22 kg/m^2^)	0.627	0.321–1.225	0.172			
Tumor location (lower vs. upper/middle)	0.567	0.308–1.044	0.068			
Neoadjuvant chemotherapy (present vs. absent)	2.356	1.251–4.434	0.008**	1.713	0.769–3.814	0.187
Differentiation (poor vs. well/moderate)	0.750	0.333–1.687	0.484			
Invasion depth (pT2/3/4 vs. pT1)	3.753	1.879–7.498	< 0.001***	0.793	0.297–2.117	0.644
Lymph node metastasis (present vs. absent)	5.372	2.384–12.106	< 0.001***	2.474	0.978–6.258	0.056
Lymphatic involvement (present vs. absent)	9.772	2.347–40.270	0.002**	1.678	0.263–10.694	0.584
Vascular involvement (present vs. absent)	8.554	2.641–27.704	< 0.001***	4.059	0.838–19.669	0.082
FSP1 (positive vs. negative)	2.379	1.223–4.629	0.011*			
GPX4 (positive vs. negative)	3.077	1.658–5.713	< 0.001***			
FSP1 and GPX4 (double positive vs. other)	5.677	2.655–12.139	< 0.001***	3.631	1.628–8.099	0.002**

*FSP1* ferroptosis suppressor protein 1, *GPX4* glutathione peroxidase 4

*p < 0.05; **p < 0.01; ***p < 0.001

### Degree of cell death depends on FSP1 or GPX4 expression level

**Fig. 3:**
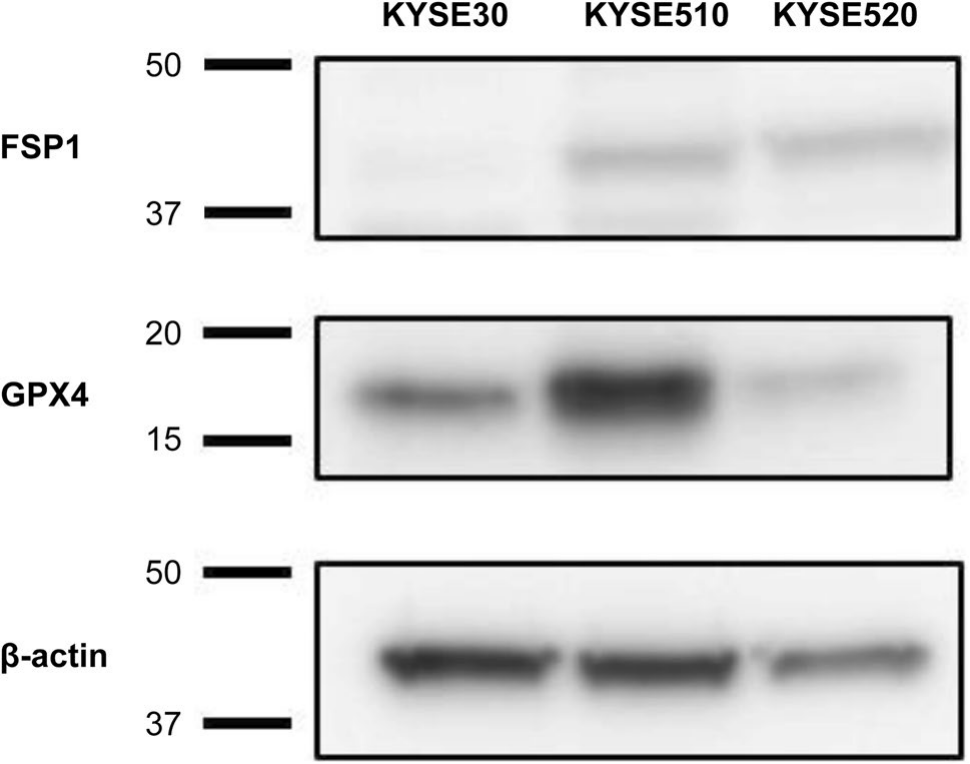
Expression of FSP1 and GPX4 in ESCC cell lines. *ESCC* esophageal squamous cell carcinoma, *FSP1* ferroptosis suppressor protein 1, *GPX4* glutathione peroxidase 4

We used three human ESCC-derived cell lines to examine whether FSP1 and GPX4 inhibition induce cell death in ESCC cells. The intensities of FSP1 and GPX4 expression assessed by Western blotting varied between the three cell lines, with negative FSP1 expression in KYSE30 and low expression in KYSE510 and KYSE520. All three cell lines expressed GPX4, as follows: moderate expression in KYSE30, high in KYSE510, and low in KYSE520 (Fig. 3).

**Fig. 4:**
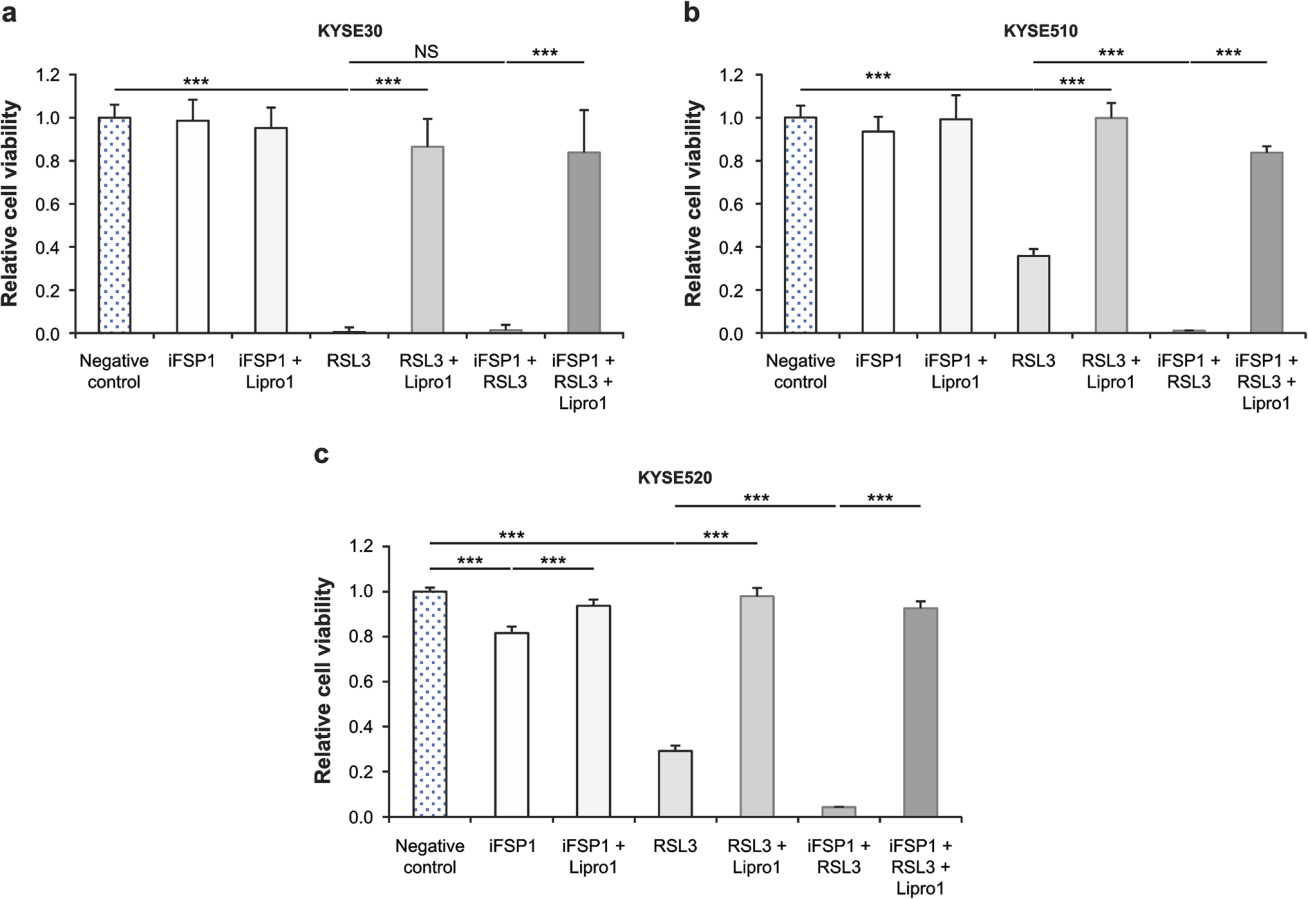
iFSP1 and RSL3 administered alone or in combination with examination of the effect adding Lipro-1 on the decrease in cell viability and the inhibition of the decrease in cell viability. *p < 0.05; **p < 0.01; ***p < 0.001. **a** KYSE30, **b** KYSE510, **c** KYSE520. *FSP1* ferroptosis suppressor protein 1, *FSP1 inhibitor* iFSP1, *GPX4* glutathione peroxidase 4, *GPX4 inhibitor* RSL3, *Liproxstatin-1* Lipro-1, *NS* not significant

To examine the correlations between the differences in FSP1 and GPX4 expression and the degree of ferroptosis induced by inhibiting FSP1 and GPX4, we administered the FSP1 inhibitor iFSP1, the GPX4 inhibitor RSL3, and the ferroptosis inhibitor Lipro-1 to each cell line and evaluated cell viability. Administration of iFSP1 alone had no effect on cell death in KYSE30, which showed negative FSP1 expression (Fig. 4a). In KYSE510 and KYSE520, both of which expressed FSP1, cell viability tended to decrease in KYSE510 (Fig. 4b) and significantly reduced in KYSE520 (Fig. 4c). Administration of RSL3 (GPX4 inhibitor) alone caused significant cell death in all cell lines (Fig. 4a–c). The iFSP1 and RSL3 combination induced cell death more potently in KYSE510 and KYSE520 than with their administration alone (Fig. 4b, c). In all dosing patterns, cell death caused by iFSP1 and RSL3 was canceled by the addition of Lipro-1 (ferroptosis inhibitor) (Fig. 4a–c).

### FSP1 and GPX4 inhibitor-induced cell death caused by ferroptosis

**Fig. 5:**
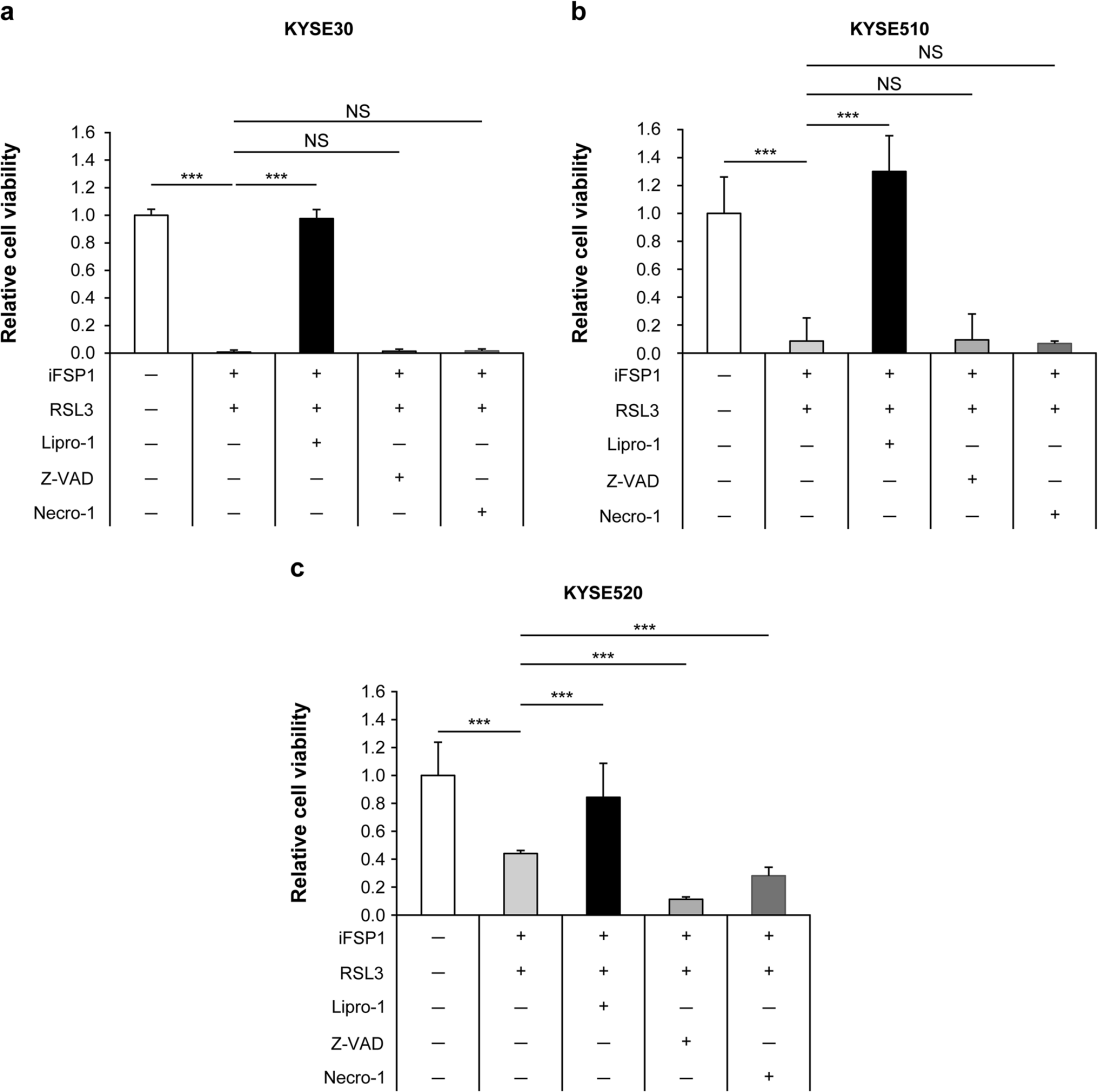
Examination of the different effects of the combination of iFSP1 and RSL3 plus Lipro-1, Z-VAD, and Necro-1 on the three cell lines in preventing the reduction of cell viability. *p < 0.05; **p < 0.01; ***p < 0.001. **a** KYSE30, **b** KYSE510, **c** KYSE520. *FSP1* ferroptosis suppressor protein 1, *FSP1 inhibitor* iFSP1, *GPX4* glutathione peroxidase 4, *GPX4 inhibitor* RSL3, *Liproxstatin-1* Lipro-1, *Necrostatin-1* Necro-1, *Z-VAD-FMK Caspase Inhibitor VI* Z-VAD, *NS* not significant

Finally, we tested whether cell death caused by the combination of iFSP1 and RSL3 could be counteracted by other cell death inhibitors. In all cell lines, cell death was markedly inhibited by the combination of Lipro-1, but administration of Z-VAD and Necro-1 did not cancel the decrease in cell viability (Fig. 5). These results indicated that cell death induced by iFSP1 and RSL3 was due to ferroptosis and not apoptosis or necrosis.

## Discussion

This study showed that expression of FSP1 and GPX4, identified as inhibitors of ferroptosis, correlated with prognosis for patients with ESCC. Furthermore, regulation of these factors markedly induced ferroptosis in ESCC cell lines. To the best of our knowledge, this report is the first to show a correlation between the combination of FSP1 and GPX4 and prognosis in ESCC.

Immunostaining of ESCC and analysis of clinical data indicated that high FSP1 and GPX4 expression was a significantly poor prognostic factor. Both FSP1 and GPX4 are capable of suppressing iron-dependent lipid peroxidation reactions [11, 12]. Oxidative stress is known to induce cell death [16], and the present results are consistent with the evidence. However, previous literature reported better prognosis in lung squamous cell carcinoma when both FSP1 and GPX4 were highly expressed compared with other expression patterns, although no difference in prognosis was observed when FSP1 or GPX4 were analyzed alone [17].

The expression pattern of GPX4 also differs depending on the cancer type. For example, GPX4 expression is reported to be upregulated in hepatocellular carcinoma and colorectal cancer [18, 19], and high GPX4 expression is associated with poor prognosis in gastric cancer [9] and lung adenocarcinoma [10]. Conversely, GPX4 expression is weaker in pancreatic cancer than in nontumor areas [20]. The reason for the conflicting expression patterns in different types of cancer is unclear, but reactive oxygen species affect cancer development in seemingly contradictory ways—promoting tumorigenesis or causing cell death, depending on the concentration [21]. Furthermore, different thresholds of reactive oxygen species concentration at which cell death occurs may be related to different levels of FSP1 or GPX4 expression in different cancers.

Ferroptosis is a mechanism of cell death that differs from apoptosis, and it is proven to have the potential to induce cell death even in apoptosis-resistant tumors [7]. Chemotherapy for esophageal cancer primarily uses apoptosis-inducing agents [3, 4]. Inhibition of GPX4 is reported to induce ferroptosis in persister tumor cells [22], the source of drug-resistant tumor cells. Therefore, cancer therapy may be improved if a novel therapy for the induction of ferroptosis is developed. In the aforementioned report showing the relationship between lung squamous cell carcinoma prognosis and FSP1 and GPX4 expression, the combination of iFSP1 and RSL3-induced marked ferroptosis, and the same was true when FSP1 and GPX4 were knocked out by CRISPR–Cas9 [17]. Furthermore, in an in vivo study, 5-aminolevulinic acid, a natural amino acid, suppressed GPX4 and resulted in tumor shrinkage [14]. Considered with the current study results, simultaneous FSP1 and GPX4 regulation may represent a new target for cancer therapy via induction of ferroptosis. Although these results were obtained only in in vitro experiments, further validation with animal experiments is needed because FSP1 and GPX4 inhibitors were simply used instead of genome editing technology, and administration in vivo is also technically simple.

The preclinical development of both iFSP1 and RSL3 has not been well reported [23]. Several reports focusing on GPX4 suppression in animals showed that mice lacking GPX4 in a nerve-specific manner developed neurodegeneration [24, 25] or died [26]. However, all studies were conducted after genome editing and cannot be directly applied iFSP1 and RSL3 administration to adult mice for whom organ development was already completed. Future studies must confirm the tumor suppression effect and any adverse events in animal experiments.

This study had several limitations. First, it was a single-center study, and the number of samples used was small. Second, the study did not examine the effects of iFSP1 and RSL3 on normal cells. Third, this study contained only a cellular experiment and not an in vivo evaluation.

## Conclusion

Overexpression of FSP1 and GPX4, especially in cases of simultaneous overexpression, is a significant poor prognostic factor in ESCC tumors. In ESCC cell lines, simultaneous suppression of both FSP1 and GPX4 caused potent cell death, which was markedly abrogated by ferroptosis inhibitors. These results indicate that simultaneous regulation of FSP1 and GPX4 may be a new target for therapy in patients with ESCC.

## Supplementary Information

Below is the link to the electronic supplementary material.

Supplementary file 1 (DOCX 32 KB)

Supplementary file 2 (TIF 590 KB)

## Data Availability

The datasets generated during and/or analysed during the current study are available from the corresponding author on reasonable request.
