# Supplementary material for: Simultaneous regulation of ferroptosis suppressor protein 1 and glutathione peroxidase 4 as a new therapeutic strategy of ferroptosis for esophageal squamous cell carcinoma
Source: Esophagus. 2022 Dec 28;20(3):492–501. doi: 10.1007/s10388-022-00982-x (PMC10234947; doi:10.1007/s10388-022-00982-x)
Supplement: Supplementary file 1 — (DOCX 32 KB) [file 10388_2022_982_MOESM1_ESM.docx]

**Title: Simultaneous Regulation of Ferroptosis Suppressor Protein 1 and Glutathione Peroxidase 4 as a New Therapeutic Strategy of Ferroptosis for Esophageal Squamous Cell Carcinoma**

**Journal name: *Esophagus***

**Wataru Miyauchi¹, Yuji Shishido¹, Yoshiaki Matsumi², Tomoyuki Matsunaga¹, Masahiro Makinoya¹, Shota Shimizu¹, Kozo Miyatani¹, Teruhisa Sakamoto¹, Yoshihisa Umekita^3^, Toshimichi Hasegawa¹, and Yoshiyuki Fujiwara¹**

^1^ Division of Gastrointestinal and Pediatric Surgery, Department of Surgery, Faculty of Medicine, Tottori University, Yonago, Japan

^2^ Division of Chemical Biology, Technical Department, Tottori University, Yonago, Japan

^3^ Division of Organ Pathology, Department of Pathology, Faculty of Medicine, Tottori University, Yonago, Japan

**Corresponding Author**

Wataru Miyauchi, MD

Division of Gastrointestinal and Pediatric Surgery, Department of Surgery, School of Medicine, Faculty of Medicine, Tottori University, 36-1, Nishi-cho, Yonago, Tottori, 683-8504, Japan

Email: [wmiyauchi@tottori-u.ac.jp](mailto:wmiyauchi@tottori-u.ac.jp)

**Online Resource**

**MATERIALS AND METHODS**

***List of antibodies and reagents***

The primary antibodies that we used were FSP1 (20886; ProteinTech Group, Chicago, IL, USA), GPX4 (ab125066; Abcam, Cambridge, UK), and β-actin (ACTB) (47778; Santa Cruz Biotechnology, Dallas, TX, USA). RPMI 1640 medium was purchased from Thermo Fisher Scientific (MA, USA). Moreover, we purchased 10% fetal bovine serum from Cosmo Bio (Tokyo, Japan) and dimethyl sulfoxide (DMSO) from Nacalai Tesque (Kyoto, Japan). We also purchased 1 × protease inhibitor and Phosphatase Inhibitor Cocktail I from Roche (Basel, Switzerland). Furthermore, Bradford Protein Assay was purchased from Bio-Rad (CA, USA) and the Cell Counting Kit-8 (CCK8) was purchased from Dojindo (Tokyo, Japan).

FSP1 and GPX4 inhibitors were iFSP1 (7162; R&D System, Minneapolis, MN, USA) and (1S, 3R)-RSL3 (RSL3; 19288; Cayman Chemical, Michigan, USA), respectively. Moreover, liporoxstatin-1 (Lipro-1; 17730; Cayman Chemical, Ann Arbor, MI, USA) was used to inhibit ferroptosis, Z-VAD-FMK Caspase Inhibitor VI (Z-VAD; S8102; Selleckchem, Houston, TX, USA) was used to inhibit apoptosis, and necrostatin-1 (Necro-1; n9037; Sigma-Aldrich, St Louis, MO, USA) was used to inhibit necrosis.

***Immunohistochemical analysis***

Immunohistochemical analyses were performed according to standard protocols. Briefly, formalin-fixed and paraffin-embedded tissue samples were processed into 4-µm thick slices. After deparaffinizing the tissue blocks, we retrieved the antigens using a microwave oven for 15 minutes in 10-mM citrate buffer (pH: 6.0). The tissue blocks were then subjected to endogenous peroxidase blockade using 3% hydrogen peroxide for 30 minutes and 10% Block-Ace (DS Pharma Biomedical, Osaka, Japan) for 30 minutes. Primary antibodies against FSP1 or GPX4 were used at dilutions of 1:1000 and 1:400, respectively. The specimens were incubated with primary antibodies overnight at 4°C. The slides were exposed to the secondary antibody, EnVision (DAKO, Tokyo, Japan), and they were counterstained using hematoxylin. Color development was performed using DAB Peroxidase Substrate Kit (Vector Laboratories, Newark, CA, USA).

***Cell lines and cell culture***

Three human esophageal squamous cell carcinoma cell lines—KYSE30, KYSE510, and KYSE520—were purchased from the Japanese Collection of the Research Bioresources Cell Bank (Ibaraki, Japan)

Clinical information on these cell lines is summarized in Table S1.

***Western blot analysis***

The cells were lysed in RIPA buffer containing 1 × protease inhibitor, and Phosphatase Inhibitor Cocktail I. We determined the protein concentrations using the Bradford protein assay. Proteins were separated by 12% Mini-PROTEAN TGX Precast Gels (Bio-Rad, Hercules, CA, USA). They were then transferred to a 0.2-µm polyvinylidene difluoride membrane (Bio-Rad). After blocking for 1 hour at room temperature with ECL Prime Blocking Agent (Cytiva, Tokyo, Japan), the membranes were reacted with primary antibodies against FSP1 (1:500) and GPX4 (1:1000) for 16 hours at 4°C. The primary antibody against β-actin (1:3000) (Santa Cruz Biotechnology) was used for normalization. Peroxidase-linked secondary antibodies against mouse and rabbit (both from Cytiva) were reacted for 1 hour at room temperature to detect protein-antibody binding. The protein signals were detected using ECL Prime Western blotting detection reagents (Cytiva) and quantified using the ImageQuant 500 (Cytiva).

***Cell proliferation assay after treatment with GPX4 or FSP1 inhibitors***

The KYSE30, KYSE510, and KYSE520 cell lines were seeded in 96-well plates (5 × 10³ cells/well) and incubated overnight. After 24 hours, the cell lines were treated with DMSO, iFSP1, or RSL3. After another 24 hours, CCK8 was added, and the cells were incubated for 1 hour. Absorbance was measured using Infinite F50 (TECAN, Männedorf, Switzerland); following this, we assessed cell viability. Moreover, the same protocol was used to investigate the synergistic effect of iFSP1 and RSL3. Each experiment was repeated three times. Drug concentrations were as follows: DMSO, 0.1%; iFSP1, 1 µM; and RSL3, 1 µM.

***Cell death inhibition assay***

The procedure of this assay and the time elapsed from cell seeding, drug addition, and cell survival evaluation were the same as those previously described. The added drugs were a combination of iFSP1 and RSL3 plus Lipro-1, Z-VAD, or Necro-1, respectively. Each experiment was repeated three times. The drug concentrations were as follows: DMSO, 0.1%; iFSP1, 1 µM; RSL3, 1 µM; Lipro-1, 1 µM; Z-VAD, 10 µM; and Necro-1, 1 µM.

**Supplementary figure legends**

**Fig. S1** Kaplan–Meier survival curves of patients with ESCC and ≥T2 or lymph node metastasis

(a, b) Survival based on the simultaneous expression of FSP1 and GPX4 in patients with ESCC and ≥T2 or lymph node metastasis.

**Table S1**. Clinical information on esophageal squamous cell carcinoma cell lines used in the study

| Cell Line | Sex | Age | Tissue for Primary Cancer | Tumor Stage | Differentiation |
| --- | --- | --- | --- | --- | --- |
| KYSE30 | Male | 64 | Esophagus | Unknown | Well differentiated |
| KYSE510 | Female | 67 | Esophagus | T4N0M1, stage 3 | Well differentiated |
| KYSE520 | Female | 58 | Esophagus | T3N0M0, stage 2 | Moderately differentiated |

**Table S2**. Neoadjuvant chemotherapy and the expression of FSP1 and GPX4 in patients with advanced esophageal squamous cell carcinoma

|  | FSP1 | | p value |  | GPX4 | | p value |
| --- | --- | --- | --- | --- | --- | --- | --- |
|  | Positive | Negative |  |  | Positive | Negative |  |
| Neoadjuvant chemotherapy |  |  | 0.772 |  |  |  | 0.614 |
| Absent | 7 | 16 |  |  | 13 | 10 |  |
| Present | 11 | 34 |  |  | 22 | 23 |  |
