# Supplementary figures and images for: Simultaneous regulation of ferroptosis suppressor protein 1 and glutathione peroxidase 4 as a new therapeutic strategy of ferroptosis for esophageal squamous cell carcinoma
Source: Esophagus. 2022 Dec 28;20(3):492–501. doi: 10.1007/s10388-022-00982-x (PMC10234947; doi:10.1007/s10388-022-00982-x)

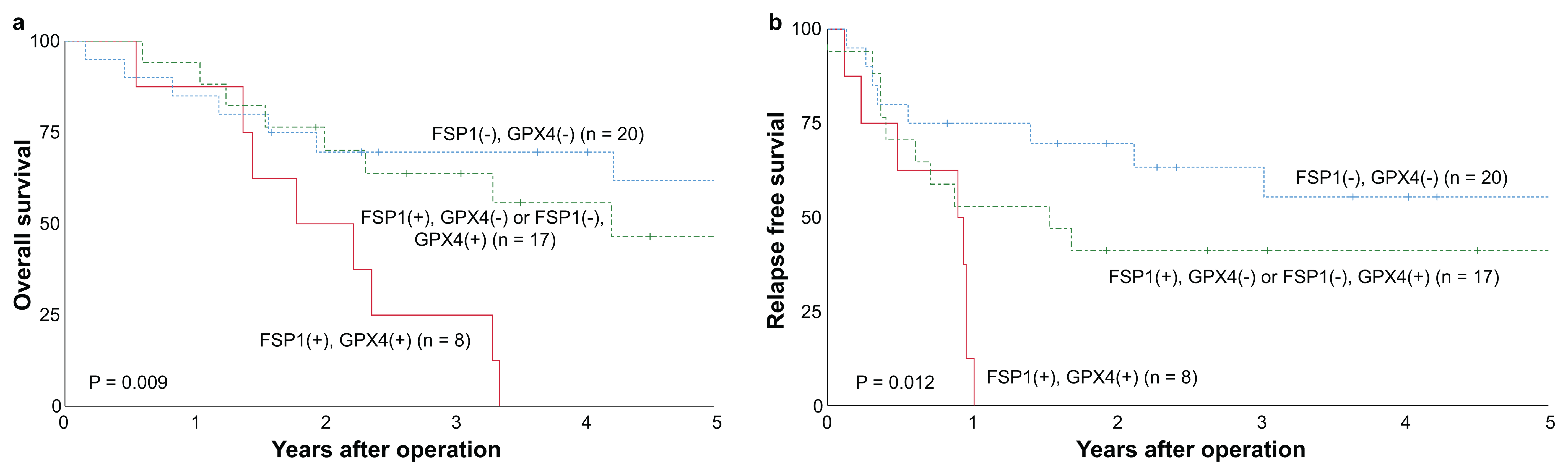

Supplement: Supplementary file 2 — (TIF 590 KB) [file 10388_2022_982_MOESM2_ESM.tif]
